# Evaluation of a corticotropin releasing hormone type 1 receptor antagonist in women with posttraumatic stress disorder: study protocol for a randomized controlled trial

**DOI:** 10.1186/1745-6215-15-240

**Published:** 2014-06-21

**Authors:** Boadie W Dunlop, Barbara O Rothbaum, Elisabeth B Binder, Erica Duncan, Philip D Harvey, Tanja Jovanovic, Mary E Kelley, Becky Kinkead, Michael Kutner, Dan V Iosifescu, Sanjay J Mathew, Thomas C Neylan, Clinton D Kilts, Charles B Nemeroff, Helen S Mayberg

**Affiliations:** 1 Department of Psychiatry and Behavioral Sciences, Emory University School of Medicine, 12 Executive Park Drive NE, 3rd Floor, Atlanta, GA, USA; 2 Max Planck Institute of Psychiatry, Munich, Germany; 3 Atlanta Veterans Affairs Medical Center, Decatur, GA, USA; 4 Department of Psychiatry and Behavioral Sciences, University of Miami Miller School of Medicine, Miami, FL, USA; 5 Department of Biostatistics and Bioinformatics, Rollins School of Public Health, Emory University, Atlanta, GA, USA; 6 Department of Psychiatry, Mount Sinai School of Medicine, New York, NY, USA; 7 Menninger Department of Psychiatry & Behavioral Sciences, Mental Health Care Line, Michael E Debakey VA Medical Center Baylor College of Medicine, Houston, TX, USA; 8 Department of Psychiatry, University of California, San Francisco & the San Francisco Veterans Affairs Medical Center, San Francisco, CA, USA; 9 Psychiatric Research Institute, University of Arkansas for Medical Sciences, Little Rock, AR, USA

**Keywords:** Clinical Research Protocol, Drugs, Investigational, HPA Axis, Anxiety disorders, Fear-potentiated startle, Fear conditioning, Neuropsychological tests, Placebo, Psychophysiology, CRH

## Abstract

**Background:**

Pharmacologic treatment options for posttraumatic stress disorder (PTSD) are limited in number and effectiveness. Medications currently in use to treat PTSD were originally approved based on their efficacy in other disorders, such as major depression. Substantial research in PTSD suggests that increased activity of corticotropin releasing hormone (CRH)-containing circuits are involved in the pathophysiology of the disease. This Phase II trial aims to evaluate the efficacy of a CRH type 1 receptor (CRHR1) antagonist in the treatment of PTSD.

**Methods/design:**

Currently untreated adult women, ages 18 to 65 years, with a primary psychiatric diagnosis of PTSD of at least 3 months’ duration, are being enrolled in a parallel-group, double-blind, placebo-controlled, randomized clinical trial evaluating the efficacy and safety of GSK561679, a novel CRHR1 receptor antagonist. GSK561679 (or matching placebo) is prescribed at a fixed dose of 350 mg nightly for six weeks. The primary trial hypothesis is that GSK561679 will reduce symptoms of PTSD, as measured by the Clinician-Administered PTSD Scale (CAPS), significantly more than placebo after six weeks of treatment. Putative biological markers of PTSD which may influence treatment response are measured prior to randomization and after five weeks’ exposure to the study medication, including: fear conditioning and extinction using psychophysiological measures; variants of stress-related genes and gene expression profiles; and indices of HPA axis reactivity. In addition, the impact of PTSD and treatment on neuropsychological performance and functional capacity are assessed at baseline and after the fifth week of study medication. After completion of the six-week double blind treatment period, subjects enter a one-month follow-up period to monitor for sustained response and resolution of any adverse effects.

**Discussion:**

Considerable preclinical and human research supports the hypothesis that alterations in central nervous system CRH neuronal activity are a potential mediator of PTSD symptoms. This study is the first to assess the efficacy of a specific antagonist of a CRH receptor in the treatment of PTSD. Furthermore, the biological and neuropsychological measures included in this trial will substantially inform our understanding of the mechanisms of PTSD.

**Trial registration:**

Clinicaltrials.gov Identifier: NCT01018992.

Registered 6 November 2009. First patient randomized 14 January 2010.

## Background

Posttraumatic stress disorder (PTSD) is a chronic and prevalent anxiety disorder that follows exposure to an overwhelming traumatic event [1]. The majority of patients with PTSD also meet criteria for other psychiatric disorders and experience significant functional impairment [2]. Many also attempt suicide [3]. Despite its impact on society, there is only partial understanding of the etiology or pathophysiology of this disorder. Effective pharmacotherapy options remain limited, with only sertraline and paroxetine approved by the US Food and Drug Administration (FDA) for PTSD. Response rates to these selective serotonin reuptake inhibitors (SSRIs) rarely exceed 60%, and even fewer patients (20 to 30%) achieve clinical remission [4]. The Institute of Medicine’s review of treatments for PTSD concluded that there is insufficient evidence to support the use of any medication for PTSD [5]. Thus, there is a clear need to develop novel and improved therapeutics for PTSD.

A growing body of literature suggests that stress-related disorders such as PTSD are associated with chronically increased activity of central nervous system circuits that utilize the 41 amino acid peptide neurotransmitter corticotropin releasing hormone (CRH, also known as corticotropin releasing factor, CRF). CRH mediates the neuroendocrine, immune, autonomic, and behavioral responses to stress [6]. CRH levels are increased by stress, resulting in activation of the hypothalamic-pituitary-adrenal (HPA) axis and increased release of cortisol and other adrenal steroids. In addition, extrahypothalamic neurons containing CRH are located throughout the brain, including the prefrontal and cingulate cortices, central nucleus of the amygdala, bed nucleus of the stria terminalis, nucleus accumbens, periaqueductal gray, and brain stem nuclei such as the major norepinephrine-containing nucleus, the locus ceruleus, and the serotonin nuclei in the dorsal and median raphe [7]. PTSD has been associated with abnormalities in HPA axis hormones, both basal and after low-dose dexamethasone dosing [8].

Increased activity of CRH-containing neurons in the amygdala activates fear-related behaviors [9], while neocortical CRH may reduce reward expectation [10]. Intracerebroventricular administration of CRH produces anxiety-like behaviors in animal models, including features particularly relevant to PTSD such as sleep disturbance, enhanced acoustic startle response, and increased conditioned fear responses. CRH type 1 receptor (CRHR1) antagonists reverse stress-related behaviors in preclinical models, including the elevated plus-maze, defensive withdrawal, light/dark transfer, and conditioned fear paradigms [11]. Animal models of early life stress have been associated with hyperactivity of CRH neurons, with chronic activation of CRHR1 in limbic brain regions [12,13]. Mice deficient in CRHR1 display decreased anxiety-like behavior and an impaired stress response [14]. Startle reactivity in mice depends on reciprocal interactions between CRH and norepinephrine systems [15]. In PTSD patients, increased cerebrospinal fluid CRH concentrations were found with single lumbar puncture sampling [16,17] as well as throughout a 24-hour period [18]. In addition, PTSD has been associated with increased psychophysiological responses, such as startle, skin conductance, and heart rate reactivity [19]. Recent studies using fear conditioning methods have found that PTSD patients have impaired inhibition of fear [20] and deficits in fear extinction [21].

Animal models suggest that CRHR1 antagonists may have therapeutic utility in stress-related disorders, but these agents have not previously been investigated in patients with PTSD [22]. Functional activity and *in vitro* binding assays indicate that GSK561679 is a potent CRHR1 antagonist. GSK561679 is an investigational drug and is not currently FDA-approved for any indication. The most frequently reported adverse event (AE) in prior studies of GSK561679 in healthy controls and depressed subjects was headache. Other commonly reported AEs included fatigue, somnolence, dizziness, nausea, nasal congestion, upper respiratory tract infection, influenza and acne. No specific laboratory abnormalities, vital sign changes, or electrocardiographic concerns have been identified in humans to date. However, degenerative changes of the testes were observed in rats, dogs, and cynomolgus monkeys, though the change was minimal in nature (that is, reduction in sperm production) and was reversible after a period of drug withdrawal. Damage to the seminiferous epithelium was also identified. This concern has led to the exclusion of men from clinical trials using GSK561679.

This clinical investigation is part of a translational collaborative effort supported by the National Institute of Mental Health (NIMH) National Cooperative Drug Discovery/Development Groups (NCDDG) program. The NCDDG program encourages collaborations between clinical and preclinical academic researchers and industry with the goals of developing novel tools for drug development and ‘first in human, first in patient testing’, as well as facilitating partnerships between academia and industry.

In this investigation, we are conducting a four-site (Emory University, Icahn School of Medicine at Mount Sinai (MSSM), Baylor College of Medicine (BCM), and the University of California San Francisco (UCSF)/San Francisco Veterans Affairs Medical Center (SFVAMC)), six-week, randomized, double-blind, placebo-controlled, parallel-arm, fixed dose trial evaluating the efficacy, safety, and tolerability of GSK561679 in female adult outpatients with PTSD.

### AIMS

The primary aim of this study is to determine the efficacy and safety of GSK561679 in the treatment of women with chronic PTSD. Secondary aims are to assess pre- and post-treatment variables believed to have clinical and pathophysiological importance in PTSD: 1) fear conditioning and extinction; 2) hormones of the HPA axis; 3) genomics and gene expression profiles; and 4) neuropsychological functioning.

## Methods/design

### Overview

Women with chronic PTSD of at least moderate severity are randomized to six weeks of double-blind treatment with either GSK561679 or placebo in a 1:1 manner. Prior to randomization, subjects complete assessments of neuropsychological function, startle testing, and HPA axis sensitivity. These measures are repeated after five weeks on the study medication to evaluate potential mediators and moderators of clinical change. DNA for genotyping is also collected. Following the six weeks of treatment, subjects enter a one month follow-up phase to monitor for safety and durability of any clinical changes.

The study is being conducted in accord with the latest version of the Declaration of Helsinki [23]. Each site’s Institutional Review Board (IRB) approved the study design, procedures, and recruitment strategies, with Emory University serving as the lead site (Emory University IRB, IRB number 00022717; Mount Sinai School of Medicine IRB, IRB number 04-0900 0001 03; Baylor College of Medicine IRB, IRB number H-30433 and the Michael E DeBakey Veterans Affairs Medical Center Research and Development Program, ID number 12G19.HBP; University of California San Francisco IRB, and the San Francisco Veterans Affairs Research and Development Committee, IRB number 12-09929). The study is registered at Clinicaltrials.gov: NCT01018992.

### Study participants

Women 18 to 65 years old who meet *Diagnostic and Statistical Manual of Mental Disorders, 4th Edition* (DSM-IV-TR) defined criteria for current PTSD of at least three months’ duration are eligible. All interested subjects undergo telephone screening to assess preliminary eligibility, and potentially eligible subjects are then scheduled for an in-office screening visit. Subjects are paid US$50 for each study visit, excluding the screening visit.

### Study sites

The study is being performed at four academic sites in the United States: Emory University, MSSM, BCM, and UCSF.

### Screening and treatment assessments

The schedule of assessments is presented in Table 1. After providing written informed consent, study participants meet with a staff member for an initial screening interview. The results of this initial interview are then presented to a study psychiatrist, who confirms the PTSD diagnosis, evaluates exclusionary psychiatric diagnoses, and clarifies medical history and current treatment. Participants who remain eligible then complete the Structured Clinical Interview for DSM-IV (SCID) [24] administered by a trained clinical interviewer. Demographic data on age, race, ethnicity, education level, marital status, employment status, and living situation are collected using a screening intake form. Family psychiatric history is collected using a self-report form listing major Axis I diagnoses and death from suicide.

**Table 1:** Schedule of events

	**Screening and pre-randomization assessment**			**Double-blind phase**							**Follow-up**	
**Visit number**	**S1**	**V1**	**V2**	**V3**	**V4**	**V5**	**V6**	**V7**	**V8**	**V9**	**V10**	**V11**
**Week**		**−1**	**−1**	**0**	**1**	**2**	**4**	**5**	**5**	**6**	**7**	**10**
**Day**		**−7**	**−1**	**0**	**7**	**14**	**28**	**35**	**36**	**42**	**49**	**70**
Informed consent	X											
Demographics/Hollingshead	X											
Vital signs	X	X	X	X	X	X	X	X	X	X	X	X
Weight	X			X			X			X	X	X
Physical examination	X									X		
12-lead ECG	X		X				X			X		
SCID-IV	X											
Dispense study drug and diary				X	X	X	X					
Screening laboratory tests	X											
Fecal occult test		X										
Safety laboratory testing			X	X	X	X	X			X		
Pregnancy testing	X		X							X		X
Pharmacokinetics					X	X	X			X		X
Psychiatric measures												
CAPS/MADRS/CSSRS/CGI	X			X	X	X	X			X	X	X
PSS-SR/QIDS-SR	X			X	X	X	X			X	X	X
CTQ and PDS screen	X											
PSQI/BPI	X			X			X			X		X
SDS				X						X		
LES				X						X		
Safety measures												
Adverse events/Concomitant medications	X	X	X	X	X	X	X	X	X	X	X	X
PRISE	X			X	X	X	X			X		
FIBSER					X	X	X			X	X	X
Exploratory aims												
Neuroendocrine measures			X	X				X	X			
Startle testing		X						X				
Neuropsychological testing		X						X				
Genotype				X								
mRNA			X					X				

Abbreviations: BPI = Brief Pain Inventory; CAPS = Clinician-Administered PTSD Scale; CGI = Clinical Global Impression; CSSRS = Columbia Suicide Severity Rating Scale; CTQ = Childhood Trauma Questionnaire; ECG = electrocardiogram; FIBSER = Frequency, Intensity and Burden of Side Effects Rating; Hollingshead = Hollingshead 4 Factors of Social Status Scale; LES = Life Experiences Survey; MADRS = Montgomery-Asberg Depression Rating Scale; PDS = Posttraumatic Diagnostic Scale; PRISE = Patient Rated Inventory of Side Effects; PSQI = Pittsburgh Sleep Quality Index; PSS-SR = PTSD Symptom Scale Self Report; QIDS-SR = Quick Inventory of Depressive Symptoms; SCID = Structured Clinical Interview for DSM-IV; SDS = Sheehan Disability Scale.

Because many patients with PTSD have experienced multiple traumas over their lifetime, it is necessary to identify the ‘index’ trauma, defined as the trauma currently causing the greatest distress or impairment to the patient. To identify the index trauma, participants complete Parts 1 and 2 of the Posttraumatic Diagnostic Scale (PDS) [25] at the screening visit. Part 1 is a short checklist of 12 items that identifies potentially traumatizing events the patient has experienced, and Part 2 asks which of these events has troubled them most in the last month. After confirmation with the study clinician, the trauma identified in Part 2 is determined to be the primary trauma and used as the focus of the Clinician-Administered PTSD Scale (CAPS) [26], to monitor PTSD symptom severity and change throughout the trial. The CAPS is a 17-item scale that assesses each of the 17 DSM-IV PTSD symptom diagnostic criteria. Each symptom is scored separately for both its frequency and intensity. Frequency ratings are made on a 5-point scale (0 = never; 4 = daily or all the time). Symptom intensity ratings are made on a 5-point scale (0 = none or no problem with symptoms; 4 = extreme, incapacitating). The total CAPS score is the sum of the frequency and intensity scores for each item. At the screening and baseline (that is Visit 3) visits, the CAPS is scored separately for symptoms present for the past week and for the past month. Subjects must score ≥ 50 for both the past week and the past month at the screening and baseline visits to remain eligible for the study. After baseline, the CAPS is assessed only for the past week of symptoms, except for the final visit (Visit 11, one month after last dose of study medicine), when past week and past month are both scored.

Depressive symptom burden is assessed using the structured interview guide for the Montgomery-Asberg Depression Rating Scale (MADRS) [27,28], a 10-item instrument assessing symptom severity over the previous week, in which each item is scored from 0 to 6. Overall symptom burden and functional status is assessed using the Clinical Global Impression of Severity (CGI-S) and, after randomization, the CGI-Improvement (CGI-I) scale [29]. Self-reported symptoms of PTSD are captured using the PTSD Symptom Scale, Self-report (PSS-SR) [30]. The PSS-SR asks subjects to indicate the frequency of the 17 DSM-IV symptom criteria items over the past week, each rated on a 0 to 3 scale. The version of the PSS-SR used in this trial includes six additional validity assessment items, which do not contribute to the total score. Self-reported depression severity is captured using the Quick Inventory of Depressive Symptoms, Self-report (QIDS-SR) [31]. The QIDS-SR is a 16-item instrument that assesses the severity of depressive symptoms present in the past seven days, with each item scored 0 to 3.

Lifetime history of suicide attempts and suicidal ideation is assessed at screening using the Columbia Suicide Severity Rating Scale (C-SSRS) [32]. The C-SSRS is a clinician-administered measure that assesses the essential information regarding suicide (behavior, ideation, lethality and severity) and distinguishes between suicidal occurrences and non-suicidal self-injury. After the screening visit, subsequent administrations of the C-SSRS assess the time period since the most recent visit. Demographic variables and family history of psychiatric illness are collected via self-report and the Hollingshead Four Factor Index of Social Status [33]. Childhood trauma history is assessed via the Childhood Trauma Questionnaire (CTQ) [34]. Inclusion and exclusion criteria are listed in Table 2.

**Table 2:** Inclusion and exclusion criteria

**Inclusion criteria**	
1	Female outpatients between 18 and 65 years old.
2	Primary psychiatric diagnosis of DSM-IV defined Posttraumatic Stress Disorder of at least three months duration.
3	Total CAPS past week and past month scores ≥ 50 at the screening and Visit 3 (randomization) visits.
4	Able to independently understand and provide written informed consent.
5	A negative urine toxicology.
6	For women of reproductive age, use of an effective birth control method^a^ for the duration of the study or abstinence.
7	Subjects who have a history of peptic ulcer disease with known etiology must provide documentation that effective treatment was provided with full eradication of ulcers and symptoms.
**Exclusion criteria**	
1	Current participating in another clinical trial in which she is, or will be, exposed to an investigational or non-investigational drug or device, or has done so within the month prior to screening.
2	Current evidence or history of significant unstable medical illness or organic brain impairment, including stroke, central nervous system tumor, demyelinating disease, cardiac, pulmonary, gastrointestinal, renal or hepatic impairment that would likely interfere with the action, absorption, distribution, metabolism, or excretion of GSK561679.
3	Subjects who in the investigator’s judgment pose a current suicidal or homicidal risk.
4	Diagnosis of anorexia nervosa or bulimia in the past year.
5	Use of systemic corticosteroids within two weeks of the Randomization Visit.
6	Treatment with any other psychoactive medication within two weeks of Visit 1, including all antidepressants, psychoactive herbal or nutritional treatment (St Johns Wort, SAM-e), lithium, other mood stabilizers, oral antipsychotics, depot antipsychotics within 12 weeks, beta blockers, thioridazine, pimozide, opiates, anxiolytics, and sedatives (with the exception of zolpidem, eszopiclone, and zaleplon). Also any treatment with any medication that the investigator judges not acceptable for this study.
7	Current pregnancy or lactation.
8	A positive stool test for occult blood.
9	Subjects who, in the opinion of the investigator, would be noncompliant with the visit schedule or study procedures (for example, illiteracy, planned vacations, or planned hospitalizations during the study).
10	Previous treatment with CRF1 receptor antagonist.
11	Any laboratory abnormality that in the investigator’s judgment is considered to be clinically significant.
12	Current treatment with exposure-based psychotherapy that targets PTSD symptoms.
13	Current or planned litigation or other actions related to secondary gain regarding the traumatic event.
14	Any cardiac condition or ECG evidence that the investigator feels will predispose the subject to ischemia or arrhythmia.
15	ECG results indicating a QTc > 450 msec at either the Screening or Randomization Visit unless repeat ECG shows that the parameter had returned to within normal range by the Randomization Visit.

^a^Acceptable methods of birth control include: surgical sterility; postmenopausal status (defined as no menses for at least 12 months); a double-barrier method (condoms plus diaphragm); hormonal contraceptives plus single barrier (birth control pills, implants (Norplant) or injections (Depo-Provera)), intrauterine device (IUD), or abstinence.

As part of the screening assessment, participants are evaluated to ensure adequate physical health and to rule out medical causes for psychiatric symptoms. This evaluation includes: a medical review of systems, physical exam, electrocardiogram and laboratory assessments (comprehensive metabolic panel, complete blood count, thyroid-stimulating hormone level, pregnancy test, and urinalysis and urine drug screen).

In preclinical testing, GSK561679 was associated with gastric mucosal erosions indicative of a local irritancy in some rat and cynomolgus monkey studies. Thus, participants who continue to meet eligibility criteria upon completion of the screening visit are instructed to collect a stool sample to assess for the presence of occult gastrointestinal bleeding, which is tested at their next study visit. A positive test results in exclusion from the study.

### Pre-randomization assessments

#### A. Biological assessments

To better understand the pathophysiology of PTSD, and to explore potential moderators and mediators of treatment outcomes, four biological measures are assessed prior to randomization.

##### 1. Psychophysiological testing

Psychophysiological testing is performed at Visit 1, prior to randomization, and repeated at Visit 7, after five weeks of study medication. Fear-potentiated startle and skin conductance response are used to assess fear conditioning and extinction, as well as the subjects’ awareness of reinforcement contingencies in the experiment. The experimental paradigm is a conditional discrimination model (termed AX+/BX−), which is specifically designed to test fear inhibition in addition to fear learning and discrimination between danger and safety signals [35]. In this paradigm, the reinforced conditioned stimulus (CS+) referred to as AX+, is always paired with the aversive unconditioned stimulus (US), whereas the non-reinforced conditioned stimulus (CS−), referred to as BX−, is never paired with the US. The conditioning phase consists of three blocks of four trials of each type (AX+, BX−, and noise alone trials). Fear inhibition is assessed on safety transfer trials that combine elements of both CSs (known as AB). There are three AB trials in the transfer test. The extinction session occurs ten minutes after the fear inhibition test and includes AX and BX trials, but neither is paired with the US. Extinction consists of six blocks of four trials of each type.

The US is a 250 ms airblast with an intensity of 80 psi directed to the larynx as described in similar human fear conditioning studies [36,37]. The CSs used in the fear conditioning and extinction sessions are different colored shapes presented on a computer monitor. The startle probe (noise burst) is a 108-dB [A] SPL, 40-ms burst of broadband noise with a near instantaneous rise time, delivered 6 seconds after CS onset. In the reinforced trials (CS+), the airblast is delivered 0.5 seconds after the startle probe and co-terminates with the CS. The stimuli are presented using SuperLab 3.0 for Windows (Cedrus, Inc., San Pedro, CA, USA) and synchronized with the psychophysiological data acquisition using a DIO card (Measurement- Computing, Inc., Norton, MA, USA). Psychophysiological data are collected with a Biopac MP150 (Biopac Systems, Inc., Goleta, CA, USA).

The acoustic startle response (eyeblink component) is measured via electromyography (EMG) of the right orbicularis oculi muscle. Two 5 mm Ag/AgCl pre-gelled disposable electrodes are positioned approximately 1 cm under the pupil and 1 cm below the lateral canthus. All resistances are less than 6 kilo-ohms. EMG activity is acquired at a sampling rate of 1 kHz, amplified and digitized using the EMG module of the Biopac system. The peak amplitude of the EMG measured between 20 and 200 ms after startle probe onset is used as the measure of startle magnitude. The skin conductance level (SCL) is acquired at a sampling rate of 1 kHz using the GSR module of the Biopac system. Two 5 mm Ag/AgCl disposable electrodes filled with isotonic paste are attached to the hypothenar surface of the palm of the non-dominant hand. The average SCL during the three to six seconds after CS onset will be subtracted from the one second pre-CS baseline to calculate skin conductance response (SCR).

To assess subject awareness and US expectancy during each experimental session subjects respond on a response keypad (SuperLab, Cedrus Corp.) during the presentation of each trial. During both fear conditioning and extinction sessions subjects press a button marked ‘+’ if they expect a CS to be followed by the US, a button marked ‘−’ if they do not expect a CS to be followed by the US, and a button marked ‘0’ if they are uncertain of what to expect.

##### 2. Genotyping

Whole blood for DNA extraction, and for DNA and lymphoblast banking at the Rutgers Cell & DNA Repository (http://www.rucdr.org/), is collected from every consenting patient at the screening visit. DNA will be used for genotyping genetic polymorphisms, mostly single nucleotide polymorphisms (SNPs), within the CRH receptor (*CRHR1*) gene and other stress-related candidate genes that emerge from previous human or animal studies, including genes from the HPA axis, monoaminergic systems, or neurotrophic systems. The study was not powered to test for genome-wide associations; however, data from it might be useful in meta-analyses across studies.

##### 3. Gene expression profiles

Whole blood collected in Tempus blood RNA tubes (Applied Biosystems, Grand Island, NY, USA) is collected for RNA at Visit 2 prior to randomization and at Visit 7, after five weeks on the study medication. Expression of specific candidate transcripts encoding chaperone and co-chaperone proteins of the glucocorticoid receptor, using real-time PCR as well as gene-expression microarrays, will be performed. We will assess the correlation of these gene-expression patterns with other putative biomarkers (for example, the dexamethasone suppression test) to evaluate whether they can be used to predict or mediate change during treatment.

##### 4. Neuroendocrine evaluation

Salivary samples are a validated means of non-invasively estimating circadian cortisol rhythms and of monitoring the dexamethasone suppression response for use as a marker of PTSD. At Visit 1, subjects are given pre-labeled Salivette collection tubes (Sarstedt, Rommesldorf, Germany) and instructed in the sample collection process. On the morning of Visit 2, one day prior to randomization, participants collect three salivary samples: (1) immediately upon awakening (and prior to eating); (2) 30 minutes after awakening, and (3) 60 minutes after awakening. Participants come to the clinic for Visit 2 at 8 am for the venipuncture collection of plasma cortisol and ACTH levels. Upon completion of Visit 2, participants are given two more Salivettes and one pill of dexamethasone 0.5 mg. On the night of Visit 2, participants collect the fourth salivary sample at 11 pm, followed immediately by ingestion of the dexamethasone. On the morning of Visit 3, subjects collect the fifth salivary sample upon awakening and return to the clinic at 8 am for the post-dexamethasone venipuncture for plasma cortisol and ACTH levels. This two-day procedure is repeated at Visits 7 and 8 in order to assess change in the neuroendocrine measures after five weeks on study medication. Due to confounding of HPA measures stemming from altered circadian rhythms, night-shift workers do not complete this component of the study.

#### B. Neuropsychological assessments

We will assess the cognitive and functional ability of participants at Visit 1, prior to randomization, and again at Visit 7, after five weeks on study medication.

##### 1. Neuropsychological battery

The MATRICS Consensus Cognitive Battery (MCCB), excluding the Social Cognition assessment portion, is used to assess for neuropsychological (NP) impairment [38]. This assessment can detect both improvements in NP performance if any occur, but also unanticipated worsening, if any, because of the lack of floor effects in the assessment. The MATRICS Battery includes ten different standard NP tests, which are administered with standard instructions and have extensive normative standards available. The tests include: Category Fluency; Brief Assessment of Cognition in Schizophrenia Symbol Coding; Trail-Making Test, Part A; Continuous Performance Test, Identical Pairs Version; University of Maryland Letter-Number Span; Wechsler Memory Scale-III Spatial Span; Hopkins Verbal Learning Test-R; Brief Visual Memory Test-Revised; and Neuropsychological Assessment Battery Mazes.

##### 2. Performance based-measures of functional capacity

The assessment of functional capacity uses instruments previously developed for use by patients with schizophrenia: the UCSD performance-based skills assessment (UPSA) [39]. The UPSA-B is an abbreviated version of the UPSA that involves role-play tasks similar in complexity to those that a community-dwelling person is likely to encounter and can be administered in ten minutes. The UPSA-B’s two domains result in a summary score ranging from 0 to 100.

#### C. Cross-site calibration

Calibration of clinical, psychophysiological and neuropsychological measures was conducted prior to beginning enrollment at each site and periodically through the trial. The lead investigators for the psychophysiological and neuropsychological components personally trained each site’s personnel in the testing procedures and actively monitor the quality of data collection through the trial. In addition, study administration, conduct, and recruitment are addressed in monthly conference calls involving each site’s principal investigator, project leads, and associated study staff.

### Randomization

Randomization occurs at Visit 3. To prevent confounding of the biological measures by the dexamethasone dose on the night before Visit 3, baseline laboratory and ECG tests are conducted the day prior at Visit 2. At Visit 3, upon completion of the post-dexamethasone venipuncture, participants are assessed with the CAPS, MADRS, C-SSRS, and CGI-S. To be eligible for randomization, participants have to score ≥ 50 on the CAPS rating for both the past week and the past month at this visit. At this visit subjects also complete the PSS-SR, QIDS-SR, the Pittsburgh Sleep Quality Index (PSQI) [40], Brief Pain Inventory (BPI) [41], and Patient Rated Inventory of Side Effects (PRISE) [42]. Self-reported functioning is assessed using the Sheehan Disability Scale (SDS) [43]. To assess the impact of recent significant life events, participants also complete the Life Experiences Survey (LES) [44].

Participants who meet all inclusion and no exclusion criteria are eligible for randomization. Treatment assignment lists, generated using randomized permuted blocks for each site, are used at each site to ensure equal allocation across treatment groups across the sites and at any time during the study. These lists were individually printed, placed in sealed opaque envelopes by the study biostatistician, and then delivered directly to the Investigational Drug Pharmacy personnel at each site. When a participant completes all procedures necessary for randomization, the study physician at each site notifies the pharmacy personnel, who assign the patient to the treatment group indicated on the randomization list.

### Protocol treatments

GlaxoSmithKline provided the study medication and matching placebo at no cost to the study. Bulk-shipped medication received at the Emory Investigational Drug Service is shipped as needed to the other sites’ investigational pharmacies. Medication throughout the trial is fixed at 350 mg nightly. The medication is formulated as white tablets, in two strengths: 50 mg and 100 mg, dispensed in separate bottles. Subjects take three of the 100 mg and one of the 50 mg tablets nightly between 6 and 8 pm, and record their dosing dates and times in a simple diary.

Pharmacokinetic measures of GSK561679 are assessed from single sample plasma collections at Visits 4, 5, 6, 9 and 11, and stored at −80°C until analyzed. Each participant’s pharmacokinetic samples are batch processed after their study participation is completed; consequently, they will not be used to guide dosing decisions or identify non-adherent subjects during their study treatment. Results from the analyses will be unblinded after finalizing all clinical trial data to identify: 1) whether any subjects did not take the study medication, and 2) to evaluate for associations between blood concentration and clinical effects.

### Study assessment visits

Symptom severity ratings are performed at weeks 1, 2, 4, and 6 post-randomization. At each of these visits, participants complete the PSS-SR, and QIDS-SR to assess PTSD and depressive symptom severity, respectively. A qualified rater administers the CAPS (for the past week), MADRS, and C-SSRS and all subjects also meet with a study psychiatrist for assessment of CGIs, concomitant medications, and adverse events. Additional self-report measures repeated at the week 6 post-randomization visit include the BPI, PSQI, LES and SDS. Vital signs are assessed at each visit and laboratory testing, particularly for liver enzyme changes, is conducted throughout the trial according to the schedule in Table 1. Side effect burden is assessed through physician inquiry and patient completion of the PRISE and the Frequency, Intensity, and Burden of Side Effects Rating (FIBSER) [42].

### Follow-up period

All participants who complete the six-week treatment phase or who terminate the trial early will return to the clinic 7 days (Visit 10) and 1 month (Visit 11) after their last dose of study medication. At these visits, rating scales and questionnaires of symptom severity, adverse events, and changes to concomitant medications are assessed, as identified in Table 1. Subjects with clinically significant abnormal laboratory tests or ECG findings at Visit 9 have those tests repeated at these follow-up visits. Upon study completion, all participants receive assistance in transitioning to another care provider as clinically appropriate at the research site (if they choose to do so) or are referred back to their physician.

### Major endpoints

The primary outcome is mean change on the CAPS past-week score between the two treatment arms. Secondary outcomes include response and remission on the CAPS past-week score. Response is defined as ≥ 50% reduction from the baseline CAPS total score at the final on-drug study visit (V9 for completers). Remission is defined as a CAPS score ≤ 20 at the final on-drug visit.

### Data management

Case report forms are adaptations of NIMH and industry-funded clinical trial protocols in PTSD. Study data is recorded on paper forms and entered into a password-protected relational database (Microsoft Office Access 2010, Microsoft, Redmond, WA, USA). A local area network resident relational database (Microsoft Office Access 2010, Microsoft, Redmond, WA, USA) serves as the secure repository for study data. Database management is performed by the study biostatistician.

### Data analyses

#### Primary aim

##### Primary analysis

Our primary statistical analysis plan will be performed under the intent-to-treat (ITT) principle that utilizes a likelihood-based, mixed effects model that assumes any missing data meet the missing at random (MAR) assumption. Continuous variables will be fit using SAS PROC MIXED (SAS Institute Inc., Cary, NC, USA), and categorical data will be fit using the gllamm add-on to Stata [45]. Prior to model fitting, we will examine the distributions of the continuous outcome variables using visual inspection of histograms and boxplots to identify potential outliers. If variables are not normally distributed or contain outliers, then log transformations will be considered or appropriate nonparametric procedures will be substituted for the analyses stated. To address the possible increase in Type I errors due to multiple outcome variables, we have *a priori* selected a single outcome measure (CAPS total for past week) which will maintain the significance level (α) 0.05 for the primary analysis. For secondary outcome measures, we will adjust *P*-values by controlling for the false discovery rate [46] and report both unadjusted and adjusted *P*-values.

#### Assessment of confounders

We expect the randomization procedure to equalize risk factors across treatment groups; however, we will produce summary statistics to explore any unexpected differences in the values of covariates in the treatment groups. While we do not expect differences in covariates in the treatment groups, it is likely that the response groups will differ on some relevant clinical variables (for example, comorbid conditions). Thus, we will formally assess response group differences in possible covariates of interest and incorporate any covariates identified that are considered both statistically and clinically relevant as additional main effects for the analyses of response predictors and moderators.

### Site effects

Our assumption for the analysis plan and power calculations is that the protocols will be implemented with sufficient consistency to avoid significant site effects in the results. Given that the randomization will be blocked by site, there is no reason to believe that the subjects will have a different *a priori* probability of response or will be significantly different from a clinical perspective. However, we will test for site differences in all outcome measures with appropriate univariate comparisons to ensure that site is not a significant confounder. If there is evidence of significant site differences in response outcomes, we will proceed with stratified analyses in place of the proposed analyses.

#### Secondary aims

##### Response rates

We will use a mixed effects model for the binary response data (generalized linear mixed model or GLMM) where, at each time point, a subject will be classified as a responder if there is at least 50% improvement in CAPS compared to the baseline measure. The factors in the model will be the same as for testing the primary hypothesis above. This analysis is an averaged group comparison of time-point specific response rates, and thus a patient can change response categories over time. We anticipate observing a significant group-by-time interaction and will perform *post hoc* tests by time point to estimate when a significant divergence in response rates occurs.

### Depressive symptoms

We will use the same approach as for the primary analysis of the CAPS as a continuous variable as described above, except using MADRS as the dependent variable.

#### Safety and tolerability

We will statistically test the equivalence of various possible adverse events (for example, headache, insomnia) from the PRISE and FIBSER using a test of the group effect in either logistic or Poisson regression models, depending on whether the outcome is a count or a binary measure. We will also compare time to dropout as well as first adverse event in both groups using a log-rank test of Kaplan-Meier curves to determine the possible effect of adverse events on the course of treatment response.

#### Exploratory aims

Effects on treatment on additional measures of change in PTSD-related symptomatology (which are the PSS-SR, CGI, and SDS) will be analyzed with a similar approach as the CAPS. These analyses will be strictly confirmatory in nature.

### Psychophysiological testing

For the fear-potentiated startle paradigm, the mean startle magnitude for each CS type in each block will be used to calculate percent change scores with the following formula using noise alone (NA) trials as the reference:

$$\begin{array}{ll} \mathrm{Percent}\mathrm{potentiation} & =100*\left(\mathrm{CS}\;\mathrm{startle}\;\mathrm{magnitude}\right)\\ & –\;\left(\mathrm{NA}\;\mathrm{startle}\;\mathrm{magnitude}\right)\\ & /\left(\mathrm{NA}\;\mathrm{startle}\;\mathrm{magnitude}\right) \end{array}$$

Discrimination between danger and safety signals will be calculated as the difference in potentiation between CS + and CS − trials. Fear inhibition will be assessed as the difference between the reinforced trials during conditioning (AX+) and the transfer trials containing elements of CS + and CS − (which are AB trials). Finally, fear extinction will be measured as the decrease in response to the CS + during the extinction session. Given the assumption of significant individual variation in the psychophysiological measures, we will perform mixed effects linear models comparing treatment group for each fear-potentiated startle measure (conditioning, discrimination, transfer of safety, and extinction) with Block, Time (baseline versus six weeks) as the within-subjects factor and Group (GSK561679 versus placebo) as the between-subjects factor and including a random intercept. The effect of interest will be the group*time interaction, indicating a significant difference between groups in change in startle over time. Skin conductance response will be analyzed in a parallel manner. Contingency awareness will be analyzed by comparing US-expectancy ratings for each CS trial type in a parallel manner.

### Neuroendocrine testing

The difference between plasma cortisol concentrations obtained pre- and post-dexamethasone will be used to calculate cortisol suppression (post-pre). Cortisol suppression will then be compared across groups by performing a linear regression of the change in cortisol level from Day 2 to Day 1 (8 am levels), while controlling for Day 1 8 am level as a covariate. Presence of dexamethasone in the Day 2 sample will confirm that the participant has taken the drug. Because individual differences in dexamethasone bioavailability can alter post-dexamethasone cortisol suppression, plasma dexamethasone concentrations will be used as a covariate in statistical analyses. The difference between salivary cortisol peak and trough will be calculated to achieve an estimate of range, which can be compared across groups using a Wilcoxon rank sum test.

### Cognitive function

Using the computer program provided with the MATRICS battery, we will develop a composite score for use in the exploratory analyses of the role of neuropsychological variables in treatment response. This score is a standard (that is ‘t’) score, so that changes from baseline are easily interpretable in terms of their effect sizes. We will also perform additional exploratory analyses of those tests in the battery specifically shown to be sensitive to PTSD in prior studies (Hopkins Verbal Learning Test and letter-number span).

### Moderator analyses

We have *a priori* specified factors that we expect may moderate the treatment effect in PTSD subjects [47]. These include, age, type and age of trauma exposure, and past treatment history. While the study is not specifically powered to detect these effects, we will analyze treatment by subgroup interaction effects in these exploratory analyses in order to provide important information for the design of future, more definitive studies. We will determine significant moderators by specifically testing the treatment by variable interaction added to the analysis of the primary outcome. Given the limited sample size, each possible moderator will be analyzed separately.

#### Statistical power/sample size

As this is the first trial of a CRHR1 antagonist in PTSD, we do not have any pilot data from which to estimate an effect size. Our sample size calculation of 154 subjects is based on an effect size of seven CAPS points, consistent with the observed difference in the study by Davidson and colleagues of the efficacy of sertraline for PTSD [48]. We used that study’s effect size and pooled between-subjects standard deviation, and expected early termination rates, to construct our power calculation on the specific test of the treatment group-by-time (two timepoints, pre and post) interaction effect for a standard repeated measures analysis of variance [49]. We used an alpha of 0.05 for all main effects and the interaction, and calculated that a sample size of 50 completers per group allows us to detect an interaction effect of this size with a power of 0.91. With the expected dropout rate of 35%, this requires recruitment of 77 subjects per group to meet the anticipated endpoint sample size, for a total randomized sample of 154 subjects.

#### Data Safety Monitoring Board (DSMB)

Three times per year, an NIMH-constituted Data and Safety Monitoring Board (DSMB) reviews the safety data of participants and the validity and integrity of the collected data. The data reviewed by the DSMB include a summary of adverse events and abnormal laboratory results, proposed protocol revisions, and all protocol deviations.

## Discussion

This study of GSK561679 efficacy constitutes the first clinical trial of a CRHR1 antagonist for the treatment of PTSD. The wealth of preclinical and clinical data implicating both central CRH-containing circuits and HPA axis dysfunction in PTSD supports testing of candidate medications that modulate this system, particularly considering the dearth of medications demonstrated to be efficacious for PTSD. This study’s neuropsychological assessments and biological measures of genotyping, gene expression profiling, fear conditioning and extinction, and dexamethasone suppression testing will provide valuable insights into the impairments and pathophysiology of PTSD, even if the GSK561679 does not prove superior to placebo in the treatment of PTSD symptoms. These multimodal variables may also be shown to have predictive value as biomarkers of PTSD treatment response.

The NCDDG initiative funding this study includes a separate project evaluating the effects of GSK561679 on the neuroendocrine profiles and startle reactivity in female healthy control subjects (Clinicialtrials.gov identifier: NCT01059227). Upon completion of both protocols we will explore the datasets to investigate shared and distinct effects of GSK561679 across the two samples.

### Trial status

Recruitment for this trial was on-going at the time this manuscript was submitted and is expected to continue through June 2014.

## Abbreviations

ACTH: adrenocorticotropic hormone; AE: adverse event; BCM: Baylor College of Medicine; BPI: Brief Pain Inventory; CAP: Clinician-Administered Posttraumatic Stress Disorder Scale; CGI-I: Clinical Global Impression of Improvement; CGI-S: Clinical Global Impression of Severity; CRF: corticotropin releasing factor; CRH: corticotropin releasing hormone; CRHR1: corticotropin releasing hormone type 1 receptor; CS: conditioned stimulus; C-SSRS: Columbia Suicide Severity Rating Scale; CTQ: Childhood Trauma Questionnaire; DSMB: Data Safety Monitoring Board; DSM-IV-TR: *Diagnostic and Statistical Manual of Mental Disorders*, *4th Edition*; ECG: electrocardiogram; EMG: electromyography; FDA: Federal Drug Administration; FIBSER: Frequency, Intensity, and Burden of Side Effects Rating; GLMM: General Linear Mixed Model; GSK: GlaxoSmithKline; HPA: hypothalamic-pituitary-adrenal; IRB: Institutional Review Board; ITT: intent-to-treat; IUD: intrauterine device; LES: Life Experiences Survey; MADRS: Montgomery-Asberg Depression Rating Scale; MAR: missing at random; MATRICS: Measurement and Treatment Research to Improve Cognition in Schizophrenia; MCCB: MATRICS Consensus Cognitive Battery; MSSM: Mount Sinai School of Medicine; NA: noise alone; NCDDG: National Cooperative Drug Discovery/Development Groups; NIMH: National Institute of Mental Health; NP: neuropsychological; PCR: polymerase chain reaction; PDS: Posttraumatic Diagnostic Scale; PHS: Public Health Service; PRISE: Patient Rated Inventory of Side Effects; PSQI: Pittsburgh Sleep Quality Index; PSS-SR: PTSD Symptom Scale, Self-report; PTSD: Posttraumatic Stress Disorder; QIDS-SR: Quick Inventory of Depressive Symptoms - Self Report; Q-LES-Q: Quality of Life Enjoyment and Satisfaction Questionnaire; SCID: Structured Clinical Interview for DSM-IV; SCL: skin conductance level; SCR: skin conductance response; SDS: Sheehan Disability Scale; SFVAMC: San Francisco Veterans Affairs Medical Center; SNP: single nucleotide polymorphism; SSRI: selective serotonin reuptake inhibitor; UCSD: University of California San Diego; UCSF: University of California San Francisco; UPSA: UCSD Performance-based Skills Assessment; UPSA-B: UPSA-abbreviated version; US: unconditioned stimulus.

## Competing interests

In the past five years, the authors report the following:

BWD has received research support from AstraZeneca, Bristol-Myers Squibb, Evotec, Forest, GlaxoSmithKline, NIMH, Novartis, Ono Pharmaceuticals, Pfizer, Takeda, and Transcept. He has also received honoraria for consulting work with Bristol-Myers Squibb, Imedex LLC, Medavante, Pfizer and Roche.

BOR has funding from the Department of Defense, NIMH, Brain and Behavior Research Foundation (NARSAD), and McCormick Foundation and recent previous support from Transcept Pharmaceuticals. Dr. Rothbaum receives royalties from Oxford University Press, Guilford, APPI, and Emory University and received one advisory board payment from Genentech.

EBB has received grant support from Pharma-Neuroboost and is co-inventors on the following patent applications: ‘Means and methods for diagnosing predisposition for treatment emergent suicidal ideation (TESI)’. European application number: 08016477.5 International application number: PCT/EP2009/061575; ‘FKBP5: a novel target for antidepressant therapy’. International publication number: WO 2005/054500 and ‘Polymorphisms in ABCB1 associated with a lack of clinical response to medicaments’. International application number: PCT/EP2005/005194.

ED is a salaried Attending Psychiatrist in the Mental Health Service at the Atlanta Veterans Affairs Medical Center in Decatur, GA. She has received grant support from Posit Science Corporation, Bristol-Myers Squibb Company, and Ortho-McNeil Janssen Scientific Affairs.

PDH has served as a consultant to Abbvie, Boeheringer-Ingelheim, En Vivo, Forest, Genentech, Otsuka-America, Roche, Sunovion, and Takeda. He has received contract research support from Genentech.

MK has served as a consultant for St. Jude Medical Neuromodulation Division and served on the Clinical Research Advisory Committee for the Shriners Hospitals for Children.

DVI has received funding (through Icahn School of Medicine at Mount Sinai) from AstraZeneca, Brainsway, Euthymics, Neosync, Roche and Shire; and consulting fees from Avanir, CNS Response, Lundbeck, Otsuka, Servier and Sunovion.

SJM has received research funding or salary support from the Banner Family Fund, Brain and Behavior Research Foundation (NARSAD), The Brown Foundation, Inc., Bristol-Myers Squibb, Department of Veterans Affairs, Evotec, Johnson & Johnson, the Marjorie Bintliff Johnson and Raleigh White Johnson, Jr. Chair for Research in Psychiatry, and the National Institute of Mental Health. He has received consulting fees or honoraria from Allergan, AstraZeneca, Bristol-Myers Squibb, Cephalon, Corcept, Genentech, Noven, Roche, and Takeda, and has received medication from Sanofi-Aventis for a NIH sponsored study.

TCN has received research medications from Actelion and GlaxoSmithKline for studies funded by the Department of Defense and Department of Veterans Affairs and has consulted for Genentech.

CDK served on a scientific advisory meeting for Allergan, co-holds a patent on the method and devices for transdermal delivery of lithium (US 6,375,990 B1), and serves on the National Advisory Board for Skyland Trail.

CBN has received research support from the NIMH and Agency for Healthcare Research and Quality. He has served as a consultant to Xhale, Takeda and SK Pharma. He has been a stockholder in CeNeRx Biopharma, NovaDel Pharma Inc., PharmaNeuroboost, Revaax Pharma and Xhale. He has had additional financial interests in Corcept, CeNeRx BioPharma, PharmaNeuroboost, Novadel Pharma Inc., and Revaax. He has served on the scientific advisory boards of American Foundation for Suicide Prevention (AFSP); AstraZeneca, CeNeRx Biopharma, Forest Labs, Janssen/Ortho-McNeil, Mt. Cook Pharma Inc., NARSAD, NovaDel Pharma Inc., Pharma-Neuroboost, Quintiles, and the Anxiety Disorders Association of America. He has served on the Board of Directors for the AFSP, George West Mental Health Foundation, NovaDel Pharma Inc., and Mt. Cook Pharma Inc. Dr Nemeroff holds a patent on the method and devices for transdermal delivery of lithium (US 6,375,990 B1) and the method to estimate drug therapy via transport inhibition of monoamine neurotransmitters by *ex vivo* assay (US 7,148,027B2).

BK, TJ, and MEK report no competing interests.

## Authors’ contributions

BWD led the manuscript development and serves as the lead study psychiatrist. BOR contributed to the study design, recruitment, and inter-rater reliability. EBB, BK, ED, PH, TJ, and SJM all contributed to the study design and wrote sections of the manuscript related to their area of expertise. MEK and MK developed the statistical analysis plan. DVI, SM, and TN each serve as the principal investigator responsible for study conduct data collection at their respective site. CBN initially conceived the project and led the study design process. CDK contributed to the initiation of the study and had advisory input into its conduct. HSM serves as the U19 grant’s overall principal investigator. All authors edited the manuscript and approved the final version.
